# Targeting the Adipose Tissue–Liver–Gut Microbiota Crosstalk to Cure MASLD

**DOI:** 10.3390/biology12121471

**Published:** 2023-11-27

**Authors:** Daniela Gabbia, Sara De Martin

**Affiliations:** Department of Pharmaceutical and Pharmacological Sciences, University of Padova, 351131 Padova, Italy; sara.demartin@unipd.it

**Keywords:** MASLD, dysbiosis, steatotic liver diseases, adipose tissue, gut microbiota

## Abstract

**Simple Summary:**

Dysfunctional gut microbiota leads to the activation of multiple pathways in the gut and in the liver that sustain hepatic inflammation and are involved in the pathogenesis of the metabolic dysfunction-associated steatotic liver disease (MASLD). Recently, many studies investigated the role of gut dysbiosis in MASLD, with the final aim of finding novel strategies to improve liver steatosis and hepatic function. Moreover, recent evidence underlines the role of adipose tissue in sustaining hepatic inflammation during MASLD development. In this review, we focus on the novel strategies proposed to improve the alteration of gut microbiota observed in MASLD patients, with a particular insight into those known to modulate gut microbiota-associated dysfunction and to affect the complex crosstalk between the gut, the adipose tissue, and the liver.

**Abstract:**

The gut microbiota is a complex system, playing a peculiar role in regulating innate and systemic immunity. Increasing evidence links dysfunctional gut microbiota to metabolic dysfunction-associated steatotic liver disease (MASLD) due to the activation of multiple pathways in the gut and in the liver, including those mediated by Toll-like receptors (TLRs), that sustain hepatic inflammation. Thus, many efforts have been made to unravel the role of microbiota-associated dysfunction in MASLD, with the final aim of finding novel strategies to improve liver steatosis and function. Moreover, recent evidence underlines the role of adipose tissue in sustaining hepatic inflammation during MASLD development. In this review, we focus on the recently discovered strategies proposed to improve the alteration of gut microbiota observed in MASLD patients, with a particular insight into those known to modulate gut microbiota-associated dysfunction and to affect the complex crosstalk between the gut, the adipose tissue, and the liver.

## 1. Introduction

Non-alcoholic fatty liver disease (NAFLD) was recently renamed as metabolic dysfunction-associated steatotic liver disease (MASLD) following a multi-society Delphi consensus statement. This definition changes the criteria to diagnose MASLD compared to the past, since at least one cardiometabolic risk factor must be present to diagnose a patient with MASLD. In the absence of metabolic risk factors, the diagnosis is cryptogenic steatotic liver disease (SLD) [1]. About 30% of the global population is estimated to have NAFLD, and this percentage is likely to increase in the next decades. Therefore, this disease is and will be a major global health problem. MASLD is also associated with obesity, type 2 diabetes, chronic low-grade inflammation, and dysregulation of the gut microbiota [1]. Simple steatotic liver disease may also evolve into a more complex disorder, non-alcoholic steatohepatitis (NASH), now replaced by the term metabolic dysfunction-associated steatohepatitis (MASH), characterized by hepatic inflammation, sustained gut dysbiosis, and increased intestinal permeability. Thus, in recent years, many studies have focused on understanding the role of the microbiota in steatotic liver disease as well as in other chronic inflammatory processes. Addressing the microbiota to potentially cure or manage MASLD is a promising area of research and is being exploited in many ongoing studies [2]. During MASLD transition to MASH, the progressive accumulation of fatty acids (FAs) into hepatocytes, which contributes to the decline in liver function, is accompanied by increased inflammation and oxidative stress. FAs are derived from three main sources: (1) the hepatic uptake of non-esterified fatty acids from plasma, (2) de novo lipogenesis, and (3) the uptake of dietary FAs from the portal circulation. Moreover, obesity and insulin resistance sustain this energy surplus by saturating the storage capacity of adipose tissue and increasing the release of FAs into the bloodstream. Furthermore, de novo lipogenesis in MASLD patients is three times higher during fasting than in the healthy population due to an aberrant stimulation of the sterol regulatory element binding protein (SREBP) pathway [3].

During the transition from MASLD to MASH, the inflammatory and metabolic interactions occurring between the gut microbiota, liver, and adipose tissue may fuel the progression of steatosis to MASH and MASH-associated fibrosis. Thus, the adipose tissue–liver crosstalk has recently been proposed as a novel target in this context. This review focuses on the recent advances in the role of gut microbiota on the adipose tissue–liver crosstalk in the pathogenesis of MASLD/MASH, discussing the novel understanding of the microbiota dysfunction in MASLD, the role of adipose tissue–liver crosstalk, and if and how targeting the gut microbiota–adipose tissue–liver crosstalk could be exploited for MASLD management.

## 2. Gut Microbiota

The gut microbiota is a complex and diverse community of microorganisms residing in the digestive tract, including bacteria, archaea, eukaryotic microorganisms, and viruses, which helps in maintaining gut and whole-body physiology [4].

Digestion and nutrient absorption, immune system regulation, protection from pathogens, and regulation of metabolic and neurological functions are some of the different functions regulated by gut microbiota in the host [5,6,7,8]. Gut bacteria favor the absorption of certain nutrients and minerals, helping in the breakdown of complex carbohydrates and fibers that the human digestive system cannot process alone. They also play an important regulatory role in immune function by training and modulating activity exerted on the immune system, teaching the immune cells to distinguish between harmful and beneficial pathogens and providing a barrier against harmful pathogens by competing for resources and producing substances that inhibit their growth [9,10,11].

Bacteria are the most abundant members of the gut microbiota and enormous differences in their species composition are present in the population. This is due to different genetic and environmental factors including diet, age, and others. For these reasons, the microbiome is unique for each person. The human gut microbiota consists mainly of five phyla: Firmicutes, Bacteroidetes, Actinobacteria, Proteobacteria, Fusobacteria, and Verrucomicrobia [12,13]. The two phyla Firmicutes and Bacteroidetes represent 90% of the gut microbiota, accounting for 60 to 80% with the classes Clostridia, Bacilli, and Negativicutes and for 20 to 40% with the classes Bacteroidia and Sphingobacteria. The Firmicutes/Bacteroidetes ratio has regularly been used as a marker of microbiome dynamics to describe changes in gut bacteria [14]. In the review by Rinninella et al., a complete taxonomic gut microbiota composition is reported, with an exhaustive description of the genera of the above-mentioned classes [12]. Along the GI tract, there is a great variability of microbiota concentration, with a low presence in the upper intestine (10–10^3^ cells/g) and a great abundance in the colon (10^11^–10^12^ cells/g), representing about 0.2 kg of weight in a healthy adult man of 70 kg [15]. 

## 3. Understanding the Role of the Microbiota in MASLD

The dysregulation of the inter-organ crosstalk, which is crucial for controlling numerous homeostatic systems, such as energy balance, glucose metabolism, and immunity, exerts a pivotal contribution to the development of several diseases, e.g., obesity and type 2 diabetes. In recent decades, several mechanisms linking gut microbiota dysfunction to MASLD development and other associated disorders, e.g., insulin resistance, type 2 diabetes, obesity, have been described and growing attention has been devoted to exploiting the microbiota as a therapeutic target for metabolic diseases [16]. Dysregulation of the different families of bacteria leads to inflammation in the gut and other tissues, including adipose tissue, muscles, the liver, and the brain, and alters glucose and energy homeostasis [16]. One of the main regulators of gut microbiota composition is the diet since food may select some LPS-releasing bacteria. LPS is one of the main pathogen-associated molecular patterns (PAMPs), which alter the gut barrier and sustain intestinal inflammation by activating Toll-like receptors (TLRs). TLRs are pattern recognition receptors participating in host defense by recognizing PAMPs and activating innate immunity [17]. The liver is an immunotolerant organ since it is constantly exposed to gut-derived PAMPs, and TLR activation is finely regulated at multiple levels (receptor expression itself, signaling cascades, receptor compartmentalization). Besides this persistent exposure to gut-derived PAMPs, the sterile insult-associated products of damaged cells, such as the damage-associated molecular patterns (DAMPs) released when hepatic oxidative stress increases, can also alter hepatic immunotolerance [18,19]. This altered gut–liver circuit fuels the low-grade persistent hepatic inflammation that concurs to MASLD/MASH progression. Adaptive immunity is also modulated by gut microbiota since it has been demonstrated that Th1, Th2, and Th17 activation can be regulated by the metabolites secreted by selected strains of gut bacteria [20,21]. Moreover, dysregulation of innate immunity is involved in the pathogenesis of NAFLD, even though the peculiar role of the different T-cell subsets is more controversial and deserves further exploration [22].

Many factors may be responsible for the gut microbiota alteration observed in obesity and MASLD, e.g., age- and diet-related factors, pharmacological treatments, as well as the stage of liver disease and the presence of co-morbid conditions [23]. Moreover, the overlap of diabetes, metabolic syndrome, and obesity sometimes makes it difficult to unravel the contribution of each metabolic factor to microbiota dysregulation, since they co-exist in patients. It is well known that an increase in the relative abundances of *Enterobacteriaceae* in stool and endotoxemia may be observed during MASLD progression [24]. Other studies demonstrated that alcohol endogenously produced by bacteria can cause fatty liver disease, as also suggested in a study conducted on a pediatric population of NASH patients that observed an increase in alcohol-producing bacteria [25].

A study investigating the composition of gut microbiota in adult and adolescent obese patients demonstrated a unique feature in terms of ecological patterns, microbial composition, and metabolism, differentially regulated in young people and adults. Obese adolescents displayed a microbiota with a peculiar metabolic profile, mainly involved in the biosynthesis of BAs and steroid acids, the metabolism of fructose, mannose, galactose, butanoate, and pentose phosphate and glycolysis/gluconeogenesis, whereas the microbiota of normal-weight adolescents is significantly more involved in the biosynthesis and metabolism of glycan, the biosynthesis of secondary bile acids (BAs), and the metabolism of steroid hormone and lipoic acid [26].

The use of antibiotics could also impact the composition of gut microbiota in metabolic dysfunction [27]. Short-term oral administration of vancomycin reduced peripheral insulin sensitivity and bile acid dehydroxylation in patients with metabolic syndrome, besides decreasing fecal microbial diversity by reducing Gram-positive bacteria, mainly Firmicutes, and increasing Gram-negative bacteria, mainly Proteobacteria. This finding sustains a role of gut microbiota in insulin resistance development [28]. A study on cirrhotic patients analyzed before and after rifaximin therapy demonstrated a reduction in the ratio of secondary to primary bile acids (BAs), linked to a modification of gut microbiome taxa, and related to the worsening of cirrhosis [29]. This imbalance in gut microbiota composition is likely to be a possible mechanism for rifaximin-induced insulin resistance [29,30]. A pilot clinical study investigating the effect on gut microbiota and markers of hepatic inflammation, steatosis, and insulin sensitivity after a 6-month treatment with rifaximin (400 mg twice a day) failed to exert beneficial effects in NASH patients [31].

The diet plays a prominent role in gut microbiota selection since the amount and types of macronutrients, nutrients, endogenous metabolites, and food components directly modulate the gut microbiota composition in terms of both quality and quantity [32]. Dietary fibers, undigested proteins, and conjugated bile acids in the bile affect the microbiota composition and function, by selecting bacterial strains producing short-chain fatty acids (SCFAs), branched-chain fatty acids (BCFAs), or toxic compounds, including ammonia, indoles, and hydrogen sulfide, which may have either positive or detrimental impacts on the gut epithelium and mucosal immune system. Undigested proteins are mainly fermented in the distal colon into hydrogen sulfide and ammonia, both exerting detrimental effects on colonic epithelium integrity at excessive amounts, and into indole derivatives, which promote interleukin (IL)-22 production, supporting the integrity of intestinal mucosa.

Preclinical and clinical evidence suggested that IL-13 might be protective towards MASH development, by preserving metabolic functions and improving inflammation in the liver and the adipose tissue. At variance, other studies suggested a loss of gut barrier function and an enhanced hepatic fibrosis associated with IL-13, which may contribute to the progression of MASH. These conflicting results deserve further investigations to unravel the effect of IL-13 on metabolic diseases and its possible use as a therapeutic target, as recently suggested by Roeb et collaborators [33].

A study by Ponziani et al. on hepatocellular carcinoma (HCC) cohorts of NAFLD and non-NAFLD patients showed that HCC patients with NAFLD and cirrhosis displayed enhanced intestinal inflammation compared to NAFLD patients without HCC and healthy subjects, as demonstrated by the increase in fecal calprotectin, an intracellular protein of the myeloid lineage cells used as a surrogate marker of intestinal inflammation [34]. This increase was inversely correlated with fecal *Akkermansia* and *Bifidobacterium.* Moreover, they observed increased plasma levels of IL-8, IL-13, and chemokine (C-C motif) ligand (CCL)3, CCL4, and CCL5 in HCC patients associated with an activated status of circulating monocytes [34]. This was accompanied by a higher abundance of *Enterobacteriaceae* and *Streptococcus* and a reduction in *Akkermansia* in cirrhotic patients. In HCC patients, *Bacteroides* and *Ruminococcaceae* were also increased, while *Bifidobacterium* was reduced. This study suggested that in patients with cirrhosis and steatosis, the gut microbiota profile is significantly correlated with systemic inflammation and can be involved in hepatocarcinogenesis.

### Diet-Related Factors Affecting Gut Microbiota

From birth, the quality and quantity of gut bacteria are modulated by a variety of factors that may change and evolve through the years until death. Among these, environment and diet are pivotal modulators, able to affect microbiota positively or negatively. 

In general, high microbiota in terms of composition and abundance is likely to predispose to a healthy status. Many studies have aimed to define the characteristics of a “healthy” gut microbiota. The results from The Human Microbiome Project suggested that healthy individuals are characterized by high taxonomic diversity, high microbial genetic richness, and more stable core microbiome composition, even though the variability is extremely high among healthy people, thus reinforcing the concept that the gut microbiota is selected during life as a consequence of many external stimuli, including environment, lifestyle, diet, host genetics, and early microbial exposure [35,36]. 

**Figure 1:**
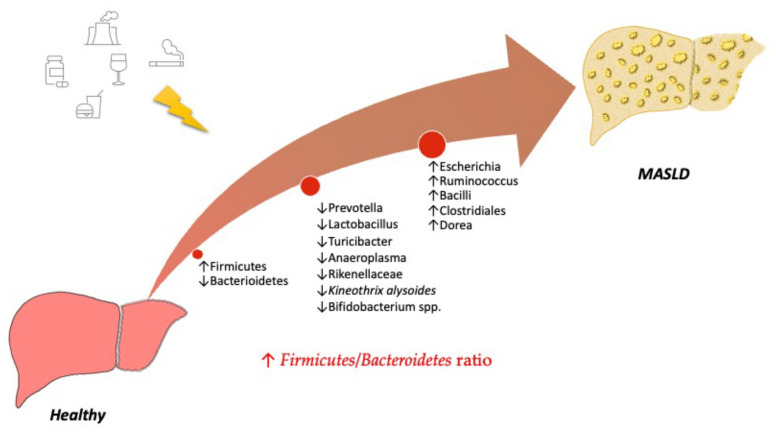
Modification in gut microbiota from a healthy to a MASLD liver.

Unhealthy diets, for example, the Western diet (WD), characterized by high consumption of fats and sugars as well as overeating, frequent snacking, and prolonged postprandial state may induce a gut microbiota dysregulation, besides the well-known effects on metabolism, e.g., hyperinsulinemia, insulin resistance, dyslipidemia, and overstimulation of the sympathetic nervous system and the renin–angiotensin system (Figure 1). Thus, diet-induced dysbiosis results in gut barrier dysfunction, increased intestinal permeability, and leakage of toxic bacterial metabolites that fuel the persistent low-grade systemic inflammation, one of the drivers of the MASLD–MASH progression. The Firmicutes/Bacteroidetes ratio is increased by high fat intake both in animals and humans [37]. Prolonged feeding (up to 80 weeks) with a high-fat diet (60% kcal from fat) in mice determines an increased relative abundance of the Firmicutes (mainly *Erysipelotrichales*, *Bacilli*, and *Clostridiales*), and consequently, the Firmicutes/Bacteroidetes ratio compared to a low-fat diet, and also an increase in *Adercreutzia*, *Coprococcus*, *Dorea*, and *Ruminococcus* [38,39]. Another meta-analysis confirmed the increase in *Dorea*, *Oscillospira*, and *Ruminococcus* abundance in obese mice. These species can ferment polysaccharides into short-chain fatty acids (SCFAs) [40]. In contrast, in obese high-fat diet (HFD)-fed mice, a drop in the relative abundance of *Turicibacter* and *Anaeroplasma* and of *Prevotellaceae* and *Rikenellaceae* belonging to the Bacteroidetes phylum was observed, together with elevated inflammation. The observed changes in the gut microbiota are similar to those observed in NAFLD patients [38,39,40,41,42]. A recently published study analyzing the gut microbiome composition in normal diet- and HFD-fed mice demonstrated a reduction in *Kineothrix alysoides* and *Turicibacter sanguinis* in the HFD group, which could be counteracted by supplementation with the bacteria [43]. HFD-related dysbiosis was also associated with a reduction in *Bifidobacterium* spp., which negatively impacted the gut barrier function [44,45]. 

Jiao and collaborators observed differentially regulated pathways in the gut microbiome of obese rodents, which were enriched in genes controlling pyruvate-related pathways, butanoate and propanoate metabolism, the pentose phosphate pathway, fatty acid biosynthesis, and glycerolipid metabolism [40]. Another study observed that HFD consumption is linked to an increase in Proteobacteria abundance, suggesting that this increase may be used as a marker for dysbiosis diagnosis and a signature of risk for disease development [46]. 

Another study observed that body weight positively correlates with Firmicutes and clostridial cluster XIVa, and negatively correlates with Bacteroidetes, linking HFD-induced changes in the gut microbiota to an obese phenotype [44].

A warning has been issued about fructose consumption and liver steatosis development. Its absorption in the small intestine, mainly due to glucose transporter-5 (GLUT5), is lesser than that of glucose [47]. As a consequence, the unabsorbed fraction in the colon can be rapidly fermented by gut bacteria into SCFAs, hydrogen, carbon dioxide, and methane, and modulate the abundance and function of colonic microbiota [48]. Thus, the fructose-induced changes in gut microbiota are mainly increased intestinal permeability and alteration of the intestinal tight junctions. The increased endotoxemia sensitizes Kupffer cells in the liver, leading to increased inflammation [49]. A recently published cross-over pilot study conducted in 10 obese subjects investigated the effect of 14 days of excessive fructose consumption and demonstrated the absence of changes in fecal microbiome, metabolome, intestinal permeability, and markers of endotoxemia, concluding that, in contrast with previous preclinical findings obtained in rodents, an excess of fructose for 14 days does not cause the gut-related modifications associated with MASLD development [48].

## 4. The Crosstalk between Adipose Tissue and the Liver during MASLD and MASH Development

The liver and adipose tissue (AT) are critical in regulating systemic energy homeostasis, and they coordinately regulate whole-body metabolism. These two organs are resilient to energy surpluses due to their capability to store excess energy in the form of triglycerides, thereby avoiding metabolic disturbances. AT is mainly composed of adipocytes, but other cell types are present, namely preadipocytes, fibroblasts, vascular endothelial cells, and immune cells. Based on their histology, function, and location in the body, two main types of AT have been described, namely the white adipose tissue (WAT) and the brown adipose tissue (BAT). White adipocytes (diameter of 20–150 μm) present in WAT have one large droplet in the center compressing the nucleus and mitochondria at one pole. In contrast, brown adipocytes (diameter of about 10–25 μm) constituting BAT have more mitochondria and small lipid droplets, which are more easily accessible than those of WAT for FFA hydrolysis and oxidation in accordance with their different function. Exposure to cold and β-adrenergic activation determine the browning of WAT, a process characterized by a phenotypic shift of white adipocytes that increase the production of the uncoupling protein-1 (UCP-1) and mitochondria metabolism. These adipocytes are also termed beige, brite, or inducible brown adipocytes, are functionally very close to brown adipocytes, and arise from specific WAT depots in response to various stimuli [50,51]. 

In humans, WAT and BAT exert different effects on the regulation of energy homeostasis that reflect their differences in morphology and location. While WAT stores surplus energy as triglycerides and releases it in the case of high energy demand [52], BAT, which represents only a minimal part of total AT and is mainly located in the supraclavicular region, is devoted to expending energy and is the site of the so-called non-shivering thermogenesis carried out by mitochondria via UCP-1 [53]. The activation of brown and beige adipocytes, generally referred to as “thermogenic adipocytes”, leads to increased triglyceride hydrolysis in AT and increased uptake of lipids and glucose from the circulation. In mammals, these processes are the consequence of sustained cold exposure and sustain heat production to maintain body temperature [51,54]. The liver may stimulate adaptive thermogenesis by the increase in BA production that promotes WAT browning [55], through the activation of the Farnesoid X receptor (FXR) and Takeda G-protein-coupled receptor-5 (TGR5). Nevertheless, conflicting results have been reported in relation to the possible role of FXR in obesity-associated metabolic disease and its inhibition and activation at the intestinal level have been shown to improve obesity, insulin resistance, and NAFLD [55]. In response to cold conditions, brown adipocytes release IL-6, insulin-like growth factor-1 (IGF1), and neuregulin-4 (NRG4), which improve glucose disposal into WAT and muscles and suppress hepatic glucose production and de novo lipogenesis, contributing to a shift from energy-consuming processes to metabolically active organs. For years, AT had been considered an inert tissue for the storing of lipids, whose only function was the thermal regulation of the body. In recent decades, many studies have highlighted the endocrine role of AT, since hormones and adipokines secreted by this tissue actively act on the regulation of many physiological processes [56]. AT exerts an endocrine function through the production of aromatases, which are involved in steroid hormone metabolism, and of several adipokines and lipokines, e.g., leptin and adiponectin, released into the circulation to modulate the metabolism and activity of target tissues and organs [57,58,59]. 

Physiologically, the adipokines adiponectin and leptin, secreted by white adipocytes, regulate the release of FFAs that can either be used for VLDL production by hepatocytes or undergo β-oxidation and ketone body production, especially after a prolonged fasting process [51]. In turn, liver-derived apolipoproteins and angiopoetin-like proteins (ANGPTLs) regulate VLDL-TG delivery to other organs and dietary lipids through intestinal-derived chylomicrons. Hydroxybutyrate and bile acids (BAs) derived from the liver exert an anti-inflammatory and insulin-sensitizing effect in WAT, and BAs stimulate thermogenesis in BAT. Moreover, several studies investigated the effect of fibroblast growth factor 21 (FGF21), a hormone-like growth factor synthesized mainly in the liver and AT that regulates lipid and glucose metabolism, in decreasing lipogenesis and increasing hepatic insulin sensitivity, even though its role is yet to be fully elucidated [60]. It has been suggested that FGF21 expression could be regulated by the carbohydrate response element-binding protein (ChREBP) produced by the liver, intestine, and adipose tissue. Besides its effect on the regulation of whole-body lipid metabolism, this protein has been demonstrated to mediate the hepatic conversion of gut microbiota-derived acetate to acetyl CoA by activating its target gene Acyl-CoA Synthetase Short Chain Family Member 2 (*Acss2*) [61].

**Figure 2:**
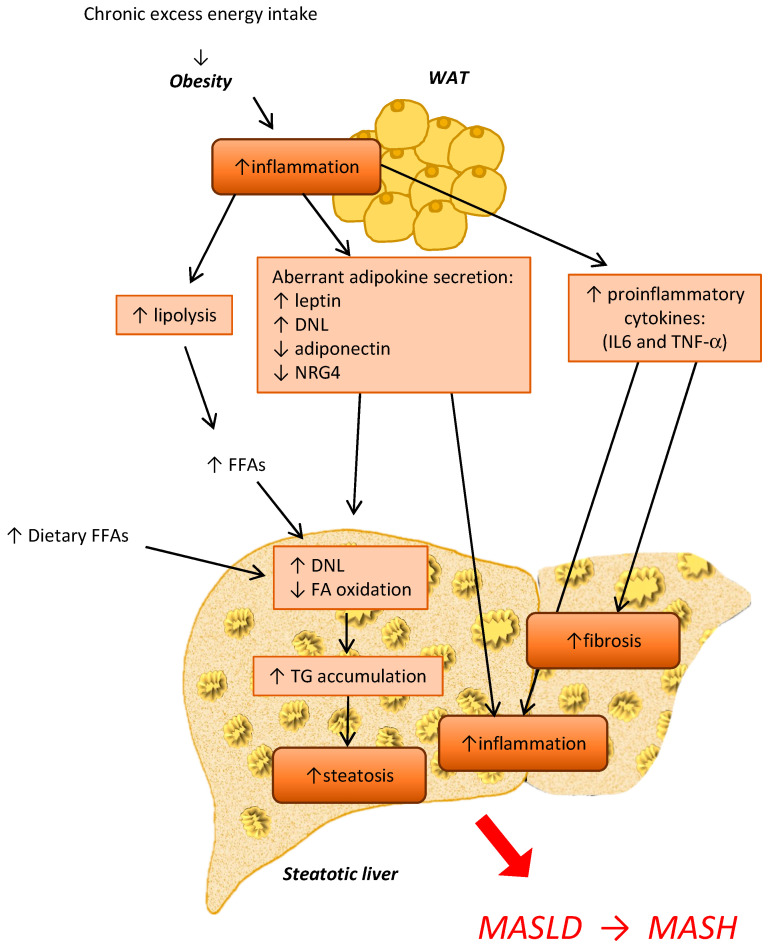
Main dysregulated pathways involved in the crosstalk between the adipose tissue and the liver during MASLD/MASH progression. Abbreviations: WAT: white adipose tissue, DNL: de novo lipogenesis, NRG4: neuregulin 4, IL-6: interleukin 6, TNF-α: Tumor necrosis factor α, FFA: free fatty acid, TG: triglycerides.

In obesity, white adipocytes release a huge amount of fatty acids (FAs) due to increased lipolysis, dysregulate adipokines and exosome secretion, and release pro-inflammatory cytokines, e.g., TNF-α and IL-6 [57]. The increase in AT lipolysis enhances circulating free FAs that are hijacked by the liver (Figure 2). This effect was also demonstrated in Abhd15-deficient mice, in which increased lipolysis led to insulin resistance and liver steatosis [62,63]. Thus, WAT dysfunction reduces the metabolic ability to respond or adapt to conditional changes, increases progressive inflammation, and determines aberrant adipokine secretion, ultimately leading to systemic insulin resistance and metabolic diseases [57]. In the liver, these processes contribute to increasing hepatic lipid content, inflammation, and induction of pro-inflammatory genes, such as fetuin-A and apolipoprotein C3, in turn triggering insulin resistance and hyperlipidemia. Therefore, the improvement of obesity-induced AT dysfunction may be targeted to reduce TG accumulation in the liver by promoting the expansion of healthy AT, WAT browning, and/or BAT activation. 

Adipokines play a multifaceted role in the modulation of the AT–liver crosstalk in MASLD. Their secretion is dysregulated in MASLD, where the increased leptin levels activate JAK2-dependent signaling pathways in the liver, through the binding to its receptor Ob-Rb in different hepatic cells, e.g., hepatocytes, Kupffer cells, and hepatic stellate cells, thus contributing to MASLD progression to MASH and fibrogenesis [64,65,66]. In contrast, the decrease in adiponectin secretion from AT occurring in steatotic patients led to reduced FA oxidation and increased de novo lipogenesis (DNL), resulting in increased hepatic TG accumulation [67]. Moreover, adiponectin has been able to alleviate diet-induced hepatic inflammation in the liver by reducing macrophage infiltration [68], and HSC activation through the activation of AMPK [69,70]. Thus, the reduction of adiponectin secretion from AT is detrimental to MASLD progression and contributes to steatosis and fibrogenesis. Other adipose tissue-derived factors may also contribute to steatosis. For example, neuregulin 4 (NRG4) is a batokine secreted by cold-activated BAT, which, being decreased in diet-induced NASH, is probably involved in hepatic inflammation and fibrosis [71,72,73,74].

Some of the mechanisms that induce the onset of MASLD concur to the accumulation of lipid droplets in AT, leading to the enlargement of adipocytes. This could overcome angiogenesis and oxygenation of AT, resulting in an inflammatory state characterized by the alteration of cytokine secretion and macrophage infiltration in AT [75,76,77]. As already stated, the gut microbiota plays a role in the modulation of appetite, intestinal permeability, nutrient absorption, and lipid and glucose metabolism. This raised interest in understanding the role of the gut microbiota in obesity and adipose tissue dysfunction [15].

### The Crosstalk between Microbiota, Adipose Tissue, and Liver

**Figure 3:**
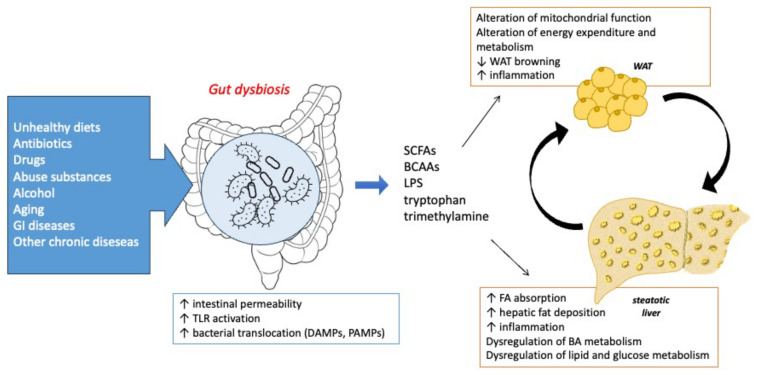
Gut dysbiosis may affect adipose tissue and liver functionality, promoting dysregulation of pathways involved in white adipocyte enlargement and fatty acid deposition in hepatocytes typically occurring in MASLD. Abbreviations: TLR: Toll-like receptor, SCFAs: short-chain fatty acids, BCAAs: branched-chain amino acids, LPS: lipopolysaccharide, BA: bile acid, WAT: white adipose tissue, FA: fatty acid, DNL: de novo lipogenesis, NRG4: neuregulin 4, IL-6: interleukin 6, TNF-α: Tumor necrosis factor α, FFA: free fatty acid, TG: triglycerides.

Gut microbiota can regulate mitochondrial function in WAT and control energy expenditure and metabolism, promoting the browning process of WAT. This regulation occurs through the activity of microbiota metabolites, e.g., short-chain fatty acids (SCFAs), LPS, BCAAs, tryptophan, and trimethylamine. As depicted in Figure 3, many factors may induce gut dysbiosis, causing an increase in intestinal permeability, TLR4 activation, and DAMP and PAMP release into the circulation, which together act on the WAT and liver crosstalk. 

In vivo studies on microbiota-depleted mice observed an increased expression of some markers of WAT browning, e.g., UCP1, peroxisome proliferator-activated receptor-γ (PPARγ), PPARγ coactivator (PGC-1α), and Cell Death-Inducing DFFA-Like Effector A (Cidea), but also an improvement of insulin sensitivity and glucose tolerance [78,79]. SCFAs, carboxylic acids like acetate, propionate, and butyrate derived from the fermentation of dietary fibers and resistant starch operated by gut bacteria are involved in the regulation of glucose and lipid metabolism and inflammation. Although SCFAs are significantly present in obese patients’ feces, their administration improved weight loss and adiposity in murine models and also in patients, due to increased WAT browning and triglyceride hydrolysis [79,80]. This effect has been exploited to improve obesity and steatosis. Butyrate seems the most promising amongst the SCFAs due to its ability to modulate energy homeostasis in both the AT and liver by increasing WAT browning and thermogenesis. As reported in the review by Amiri, the two main mechanisms of butyrate beneficial effects are its action as a histone deacetylase (HDAC) inhibitor, affecting the expression of several genes involved in multiple pathways and the ability to bind to specific G-protein-coupled receptors (GPCRs), triggering intracellular responses involved in energetic homeostasis [81]. Supplementation with butyrate was able to induce UCP1-mediated thermogenesis in BAT and WAT browning by increasing peroxisome proliferator-activated receptor γ coactivator-1α (PGC-1α), a PPARγ coactivator that regulates mitochondrial biogenesis [82,83]. 

In contrast, other metabolites produced by gut microbiota have detrimental effects on AT thermogenic function. For example, LPS, whose increase was observed in gut dysbiosis and MASLD, decreases WAT browning through TLR4 activation and modulation of FOXc2 expression, which in turn regulates the transcription of UCP1, PGC-1α, and PR domain-containing 16 [55]. Also, increased tryptophan production was linked to an increase in WAT inflammation via micro-RNA modulation in preclinical models and humans [84]. The upregulation of miR-181 in high-fat diet-fed mice, a consequence of changes in the abundance of microbial metabolites induced by gut microbiota dysbiosis, contributes to the development of obesity, insulin resistance, and WAT inflammation. Thus, the authors of this study suggest that this or other mi-RNAs may be exploited as therapeutic targets for obesity and related metabolic disorders. 

## 5. Strategies to Target the Gut Microbiota–Adipose Tissue–Liver Crosstalk for MASLD Management

In recent years, many efforts have been devoted to developing strategies to effectively improve MASLD by modulating gut microbiota. Some recent clinical trials assessing the modulation of gut microbiota in metabolic dysfunction are reported in Table 1. One of the most exploited interventions is the modification of dietary habits, i.e., the adoption of a healthier nutritional approach or the administration of nutritional supplements, e.g., prebiotics and fiber, able to modulate gut microbiota. Lifestyle modifications, like dietary restrictions and increased calorie consumption by physical activity, are the main interventions that positively affect lipid accumulation in the liver and adipose tissue expansion. [85,86]. A pilot study on 15 obese/overweight patients with MASLD demonstrated an improvement in body weight, liver fat, and systemic inflammation after 3 weeks of a Hypocaloric Hyperproteic Diet (HHD), which was accompanied by a modulation of enzymes involved in amino acid and carbohydrate metabolism and of gut microbiota. In particular, a decrease in *Lachnospira* and an increase in *Blautia* and *Butyricicoccus* was observed [87].

The term “prebiotics” refers to non-digestible nutrients, e.g., inulin-type fructans, galacto-oligosaccharides, arabinoxylan, and arabinoxylan oligosaccharides. They may exert a beneficial role on gut bacteria, thus improving host health [32]. These non-digestible fibers are contained in wheat and fungal chitin-glucan and seem to improve hepatic lipogenesis, insulin resistance, and adiposity by the transcriptional modulation of some metabolic genes. Moreover, several phenolic compounds may have multiple beneficial effects on the host by positively selecting the growth and/or activity of specific gut bacteria strains. Prebiotics are able to reinforce the mucosal gut barrier and promote the production of some gut hormones controlling appetite, glucose homeostasis, and inflammation. A recently published study evaluated the effects of fructo-oligosaccharides and galactooligosaccharides in high-fat diet-induced MALSD, observing that these two prebiotics are able to improve insulin resistance, reduce hyperglycemia, triglyceridemia, and cholesterolemia [88]. Moreover, these prebiotics effectively increased the abundance of *Bacteroides acidifaciens* and *Bacteroides dorei*, producing acetate that is likely to improve diet-induced intestinal barrier leakage, thus ameliorating hepatic lipogenic pathways and reducing inflammatory markers, such as p-NFκB, IL-6, iNOS, COX-2, TNF-α, IL-1β, and nitrotyrosine. A double-blind, randomized, placebo-controlled, phase 2 trial assessing the efficacy of a synbiotic supplement in NAFLD patients observed a modulatory effect on gut microbiota. However, this supplementation was ineffective in improving hepatic fat content or fibrosis in these patients [89].

A study by Mujico and collaborators demonstrated that HFD-induced gut dysbiosis was effectively counteracted by supplementation with an oleic acid-derived compound and a combination of n-3 fatty acids EPA and DHA that was able to modulate body weight and restore healthy gut microbiota. In particular, oleic acid-derived compounds effectively restored the proportions of bacteria, reducing clostridial cluster XIVa and Enterobacteriales and increasing Bifidobacterium spp. altered by HFD, and the EPA and DHA combination significantly increased the abundance of Firmicutes, especially the Lactobacillus group [44].

Another therapeutic strategy proposed to restore diet-associated gut dysbiosis in liver steatosis is fecal microbiota transplantation (FMT). Many ongoing clinical trials, as reported in Table 2, are investigating the effect of fecal microbiota transplants as a possible treatment for MASLD/MASH. A randomized clinical trial on 75 patients observed that FMT reduced hepatic fat accumulation due to an improvement in gut dysbiosis [90]. Nevertheless, FMT exerted a better effect on gut microbiota in “lean” patients with steatosis, and significant differences in the clinical features and gut microbiota were observed between the “lean” and “obese” groups, suggesting that FMT is more likely to have less effect in MASLD.

The results of these clinical trials will help us to better understand the role of gut microbiota dysfunction in MASLD and unravel if this could be effectively targeted to cure this disease. It should be underlined that convincing preclinical evidence supports the use of FMT and bacterial strain supplementation in reverting MASLD development [91,92,93]. 

Besides the fecal transplant, the treatment with some specific bacteria strain has also been proposed for MASLD/MASH therapy. Several in vitro and in vivo studies have suggested that “probiotics”, defined as live organisms that may beneficially affect human health, could improve intestinal permeability by competing with pathogenic bacteria, exerting immunomodulatory effects, and regulating the gut–brain axis [94]. 

The treatment with *Kineothrix alysoides*, a gut bacteria whose abundance is reduced in steatosis, significantly improved MASLD and weight loss in HFD-fed and in high-fat high-fructose-fed mice, preventing liver damage, ameliorating lipid metabolism, and counteracting diet-related gut dysbiosis [43]. 

Based on preclinical results, clinical trials have also been performed to test this approach. A randomized placebo-controlled trial on NAFLD patients treated for 8 weeks with a multi-probiotic named “Symbiter”, containing 14 probiotic bacteria of the Bifidobacterium, Lactobacillus, Lactococcus, and Propionibacterium genera, demonstrated a significant reduction in TNF-α and IL-6 levels after treatment, accompanied by an improvement of the fatty liver index and serum levels of AST and GGT [95]. A similar improvement of AST was observed in NAFLD patients treated daily with 500 million *Lactobacillus bulgaricus* and *Streptococcus thermophilus* [96]. Another multi-probiotic mixture of eight probiotic strains (*Streptococcus thermophilus*, *Bifidobacteria* [*B. breve*, *B. infantis*, *B. longum*], *Lactobacillus acidophilus*, *L. plantarum*, *L. paracasei*, and *L. delbrueckii* subsp. Bulgaricus) was tested in a placebo-controlled trial with 44 NAFLD children, confirming an improvement of NAFLD associated with a decrease in body mass index (BMI) and an increase in GLP-1 and activated GLP-1 (aGLP-1) after 4 months [97].

A study evaluating IMM-124E, an oral, non-absorbable compound containing poly-clonal anti-LPS immunoglobulins that could interact with intestinal LPS and the immune system demonstrated a dose-dependent improvement in endotoxemia and liver injury markers, namely AST, ALT, and CK 18, even though it failed to demonstrate a reduction in the fat content of the liver in NASH patients and was thus discontinued [98]. 

Bariatric surgery, a long-term effective treatment for weight control in obese patients, modulates gut microbiota and improves obesity-related comorbidities [99]. Its beneficial effect on NAFLD is due not only to weight loss and AT reduction, but also to the reduction in pro-inflammatory cytokine release and FAs reaching the liver from AT, which consequently modulate lipid and glucose metabolism [100]. Several targets, e.g., the gut-derived peptide FGF15/19 and FXR, are likely to be responsible for the favorable metabolic changes observed after bariatric surgery. In NASH patients, an analog of FGF19, aldafermin, was also effective in increasing the rare genus *Veillonella*, a commensal microbe with lactate-degrading and performance-enhancing properties, able to decrease the toxic BAs [101].

Despite the beneficial effects of bariatric surgery, recent evidence has shown that the gut microbiota is only partially recovered [102], suggesting the need for more randomized controlled trials and larger prospective studies to better identify associations between gut microbiota, obesity, and bariatric surgery. Moreover, whether the modulation of gut microbiota after bariatric surgery is a mere consequence of anatomical, hormonal, and metabolic changes or a contributor to its beneficial effects remains to be clarified.

In the absence of drugs specifically approved for MASLD/MASH treatment and due to the close correlation between type 2 diabetes and liver steatosis, some anti-diabetic drugs have been subjected to investigation in this field [103,104,105]. These drugs, including sulfonylureas, TZDs, metformin, SGLT2 inhibitors, and GLP-1 agonists, may also regulate thermogenic adipose tissue [106]. The mechanisms of action on AT involve the activation of classical non-shivering thermogenesis, the inhibition of lipolysis and lipase activity, and the promotion of glucose uptake and oxidation due to the modulatory effect on AMPK, peroxisome proliferator-activated receptor gamma (PPARγ), PPARγ coactivator-1α (PGC-1α), protein PR domain-containing 16 (PRDM16), and UCP1 pathways [106]. Moreover, metformin, GLP-1 agonists, SGLT2 inhibitors and pioglitazone have demonstrated an effect on gut microbiota, particularly by enhancing the abundance of *Akkermansia muchiniphyla* and SCFA-producing bacteria and decreasing the Firmicutes/Bacteriodates ratio and specific pathogens, e.g., *Escherichia coli* and *Salmonella* [103,107,108,109,110]. Thus, anti-diabetic drugs may have a more complex and intriguing mechanism involving the modulation of the crosstalk between the gut microbiota, adipose tissue, and the liver.

## 6. Strengths and Limitations of Current Studies

It has been estimated that MASLD may affect 17–46% of European adults, with a progressive increase due to aging [111]. MASLD is closely related to obesity (90% of obese people are affected) and type 2 diabetes (50–70% of T2D patients have MASLD). The number of MASLD patients is expected to dramatically increase in the coming years due to a variety of factors, e.g., dysregulated diet and unhealthy lifestyles. Increasing evidence suggests that the interplay between gut microbiota modifications and the liver–adipose tissue crosstalk could be targeted in MASLD.

Recently, clinical trials have investigated the possibility of modulating this crosstalk through different approaches. One of the main modulators of gut composition is the diet, thus the adoption of a healthy diet and particularly the enrichment of beneficial components, e.g., fiber and prebiotics, seems to be useful for MASLD patients. Nevertheless, the encouraging preclinical results seem to have poor translational value, and many clinical studies failed to demonstrate substantial benefit, particularly in MASH patients. Many studies are ongoing to evaluate the potential of FMT on MASLD improvement and their results are likely to give more insight into this complex issue.

One of the main challenges regarding the translational approach in this field is represented by the fact that gut microbiota composition may greatly vary among species in response to the same stimuli, thus complicating the intrinsically complex interaction between gut microbiota and host physiology.

Another point that needs further investigation is how great the modification of gut microbiota on the host metabolism and lipid dysfunctional processes observed in MASLD patients could be. It remains unclear if the modification of gut microbiome alone may be sufficient to improve a multifactorial disease like MASLD or if a combination of drugs acting on different molecular targets is likely to be more appropriate to treat this liver disease, possibly by the development of personalized treatment approaches based on each patient and one’s gut microbiota profile. Moreover, most of the studies conducted so far investigated the effect of gut microbiota modulation on the liver status without evaluating the AT and, thus, lacking important information on the AT–liver crosstalk. This aspect seems to be crucial to have a complete picture of MASLD and the mechanisms of the pharmacological and non-pharmacological interventions herein described.

## 7. Conclusions

Increasing evidence links dysfunctional gut microbiota to MASLD, due to the activation of multiple inflammatory pathways that sustain hepatic damage and transition to MASH. Many studies have been focused on unravelling the role of microbiota-associated dysfunction in MASLD to find novel treatment strategies. More recently, the role of the adipose tissue in this crosstalk was recognized and underlined. Different strategies have been proposed to modulate the microbiota-associated dysfunction in MASLD, characterized by the ability to interfere with the complex crosstalk between the gut, the adipose tissue, and the liver. Although the results of the ongoing trials have sometimes been contradictory, the modulation of this complex interplay represents a promising target for MASLD patients and deserves further attention.

## Figures and Tables

**Table 1:** Completed or terminated clinical trials evaluating therapeutic strategies to modulate gut microbiota alteration in NAFLD/NASH patients.

Study Name	Treatment	Study Population	Results	NCT Number
Rifaximin in fatty liver disease	Rifaximin tablet, 400 mg twice daily for 6 weeks	15 patients (13 male and 2 female)	No differences in ALT, peripheral glucose uptake, hepatic insulin sensitivity, hepatic lipid content from baseline after 6-week treatment. No consistent difference in relative abundance of fecal microbiota	NCT01355575
Synbiotic treatment in NAFLD (INSYTE study)	Synbiotic supplement (fructo-oligosachharide with a degree of polymerization <10 at 4 g/twice a day plus *Bifidobacterium animalis* subsp. *lactis* BB-12—10 billion CFU/day)	89 patients (58 male and 31 female)	Synbiotic sustained *Bifidobacterium* and *Faecalibacterium* abundance, at the expense of *Oscillibacter* and *Alistipes* No significant difference in liver fat after treatment Only weight loss was associated with a significant decrease in liver fat	NCT01680640
IMM-124E in NASH patients	IMM-124E, 600 or 1200 mg three times daily for 24 weeks	133 NASH patients (63 male and 70 female)	No improvement in NASH was observed although a decrease in serum LPS levels and in AST and ALT	NCT02316717
Dietary intervention and intestinal microbiota in NAFLD	Hypocaloric Hyperproteic Diet (HHD, Eurodiets^®^, ~1000 kcal/day, ~125 g protein/day) for 3 weeks	15 overweight/obese patients with NAFLD	Modification of gut microbiota composition and function was observed after 3 weeks of HHD HHD decreased body weight, liver fat, and systemic inflammation	NCT01477307

Data accessed at https://clinicaltrials.gov/search?cond=NAFLD%20-%20Nonalcoholic%20Fatty%20Liver%20Disease&term=Gut%20Microbiota&aggFilters=results:with, on 17 October 2023.

**Table 2:** Recent ongoing clinical trials investigating FMT and probiotic supplementation in NAFLD/NASH.

Study Title	Conditions	Interventions	NCT Number
Intestinal microbiota transplantation for nonalcoholic fatty liver disease	NAFLD	Intestinal microbiota transplantation	NCT03648086
The effect of consecutive fecal microbiota transplantation on NAFLD	NAFLD	Gut microbiota transplantation	NCT04465032
A prospective, randomized, controlled pilot study to characterize the intestinal microbiome and to evaluate the safety and fecal microbiome changes following administration of lyophilized PRIM-DJ2727 given orally in subjects with non-alcoholic fatty liver disease	NAFLD with history of diabetes mellitus	PRIM-DJ2727	NCT04371653
To evaluate the beneficial effect of probiotics on NAFLD patients and the role of gut microbiota modulation	NAFLD	Probiotics	NCT05402449
Impact of FMT on the phenome in patients with NAFLD and fibrosis	NAFLD	FMT	NCT06024681
Probiotics in NASH patients—PROBILIVER TRIAL	NAFLD	Probiotics	NCT03467282
Fecal microbiota transplantation for the treatment of non-alcoholic steatohepatitis	NAFLD	FMT	NCT03803540
Interest in “Combo” (a combination of dietary supplements including probiotics) in NASH improvement	NASH	Treatment with Combo (dietary supplements including probiotics)	NCT04781933
Effects of *Bacillus coagulans* on liver and gut microbiota function in NAFLD	NAFLD	*Bacillus coagulans*	NCT05635474
Effects of fecal microbiota transplantation on weight in obese patients with NAFLD	NAFLD	Diet/FMT/physical activity	NCT04594954
Soluble fiber supplementation in NAFLD	NAFLD	Fructo-oligosaccharide-enriched inulin supplement	NCT05480696
MLCT Oil for fatty liver—PASS Trial	NAFLD	MLCT Oil/LCT Oil	NCT05217745
The effect of probiotics on the clinical outcomes and gut microenvironment in patients with fatty liver	NAFLD	Probiotics (microbial cell preparation)	NCT04074889
Effect of probiotics or berberine in hepatic steatosis markers, cardiometabolic and microbiotic profile in NAFL.	NAFLD/obesity	Probiotics/berberine/probiotics and berberine	NCT05523024
Nutraceutical improvement of glucose metabolism, NAFLD and insulin resistance by oat-fiber supplementation in Type 2 diabetes mellitus patients	Type 2 diabetes/NAFLD	Drinking powder supplement	NCT05654805
Synbiotics and fecal microbiota transplantation to treat non-alcoholic steatohepatitis	NASH/NAFLD	Combination product: LFMT	NCT05821010

Data accessed at https://clinicaltrials.gov, on 17 October 2023.
